# Specificity Characterization of SLA Class I Molecules Binding to Swine-Origin Viral Cytotoxic T Lymphocyte Epitope Peptides *in Vitro*

**DOI:** 10.3389/fmicb.2017.02524

**Published:** 2017-12-18

**Authors:** Caixia Gao, Xiwen He, Jinqiang Quan, Qian Jiang, Huan Lin, Hongyan Chen, Liandong Qu

**Affiliations:** Heilongjiang Provincial Key Laboratory of Laboratory Animal and Comparative Medicine, State Key Laboratory of Veterinary Biotechnology, Harbin Veterinary Research Institute, Chinese Academy of Agricultural Sciences, Harbin, China

**Keywords:** swine leukocyte antigen, major histocompatibility complex, CTL epitope peptide, specific binding, MHC restriction

## Abstract

Swine leukocyte antigen (SLA) class I molecules play a crucial role in generating specific cellular immune responses against viruses and other intracellular pathogens. They mainly bind and present antigens of intracellular origin to circulating MHC I-restricted cytotoxic T lymphocytes (CTLs). Binding of an appropriate epitope to an SLA class I molecule is the single most selective event in antigen presentation and the first step in the killing of infected cells by CD8+ CTLs. Moreover, the antigen epitopes are strictly restricted to specific SLA molecules. In this study, we constructed SLA class I complexes *in vitro* comprising viral epitope peptides, the extracellular region of the SLA-1 molecules, and β2-microglobulin (β2m) using splicing overlap extension polymerase chain reaction (SOE-PCR). The protein complexes were induced and expressed in an *Escherichia coli* prokaryotic expression system and subsequently purified and refolded. Specific binding of seven SLA-1 proteins to one classical swine fever virus (CSFV) and four porcine reproductive and respiratory syndrome virus (PRRSV) epitope peptides was detected by enzyme-linked immunosorbent assay (ELISA)-based method. The SLA-1^∗^13:01, SLA-1^∗^11:10, and SLA-1^∗^11:01:02 proteins were able to bind specifically to different CTL epitopes of CSFV and PRRSV and the MHC restrictions of the five epitopes were identified. The fixed combination of Asn^151^Val^152^ residues was identified as the potentially key amino acid residues influencing the binding of viral several CTL epitope peptides to SLA-1^∗^13:01 and SLA-1^∗^04:01:01 proteins. The more flexible pocket E in the SLA-1^∗^13:01 protein might have fewer steric limitations and therefore be able to accommodate more residues of viral CTL epitope peptides, and may thus play a critical biochemical role in determining the peptide-binding motif of SLA-1^∗^13:01. Characterization of the binding specificity of peptides to SLA class I molecules provides an important basis for epitope studies of infectious diseases in swine, and for the rational development of novel porcine vaccines, as well as for detailed studies of CTL responses in pigs used as animal models.

## Introduction

The swine major histocompatibility complex (MHC), also referred to as swine leukocyte antigen (SLA), has been associated with the porcine immune response to various infections and vaccinations (Lunney and Murrell, 1988; Lumsden et al., 1993; Lunney et al., 2009). SLA has been mapped to pig chromosome 7, and comprises highly polymorphic classical class I, class II, and conservative class III gene clusters (Renard et al., 2006). The SLA class I gene cluster contains three constitutively expressed classical genes, *SLA-1, SLA-2*, and *SLA-3*, which are expressed by most cells. The *SLA-1* gene has the highest expression level whereas *SLA-3* has the lowest as a result of different promoter activity (Tennant et al., 2007). The *SLA-1, SLA-2*, and *SLA-3* genes are highly polymorphic, and 192 alleles (69 *SLA-1*, 87 *SLA-2*, 36 *SLA-3*) have been designated by the SLA Nomenclature Committee of the International Society for Animal Genetics in the Immuno Polymorphism Database (IPD)-MHC SLA sequence database to date^1^ [Release 2.2.0.0 (2017-06-01)] (Maccari et al., 2017). The extreme polymorphisms of SLA class I genes are concentrated in the α1 and α2 domains, which resemble each other structurally, and together form the class I heavy chain protein peptide-binding groove (PBG). Three-dimensional (3D) crystal structure analysis has indicated that the PBG contains six pockets (A–F), and epitope peptides fixed in the PBG by these pockets. The different allelic forms of SLA class I genes were confirmed to bind different classes of peptide, determined by the fit between the pockets in the PBG of the SLA complex, and the anchor residues in the peptides (Zhang N. et al., 2011; Fan et al., 2016). SLA class I heavy chain, epitope peptide, and β2-microglobulin (β2m) have also been shown to form a ternary complex, with the protein complexes being expressed constitutively on the surface of virtually all nucleated cells. These are crucially important for the normal growth of CD8+ cytotoxic T lymphocytes (CTLs), and play a pivotal role in the cell-mediated immune response against viral infections and cancer. The protein complexes are mainly involved in the adaptive immune response through their presentation of endogenous epitope peptide antigens to circulating MHC I-restricted CTLs (Neefjes et al., 2011). CTLs kill the specific target cells directly, and also induce host cell-mediated specific immune responses by simultaneously identifying the epitope peptides specifically bound to SLA class I molecules and the SLA class I molecules (MHC restriction). This represents one of the important host-mediated immune-defense responses for controlling virus infection. Furthermore, the protein complexes also interact with natural killer (NK) cells to prevent NK-mediated cytotoxicity (Kwiatkowski et al., 1999).

Pigs are important experimental animals for veterinary and medical studies, including studies of virus infections and pathogenesis, host immunological responses, vaccine evaluation, the identification of T-cell epitopes, and so on. An understanding of peptide binding to SLA class I proteins is therefore of major practical interest. In addition, pigs have an evolutionary resemblance to humans, and share anatomical, physiological, immunological, metabolic, and nutritional similarities, making them promising organ donors for xenotransplantation. The structures of human and mouse MHC class I molecules and their interaction with bound antigenic peptides along with the roles of peptide-interacting pockets in the PBG have been intensively studied. Nevertheless, only a very limited set of the SLA class I molecules have been well documented and reported (Zhang N. et al., 2011; Fan et al., 2016), and the characteristics of peptide presentation for SLA class I molecules and cellular immune mechanisms have remained elusive until now. The structure and function of SLA class I complexes constructed *in vitro* are currently used to simulate the functions of SLA class I molecules *in vivo* and numerous *in vitro* SLA class I complexes have been constructed and different peptide–SLA-I binding assays have been suggested (Oleksiewicz et al., 2002; Sylvester-Hvid et al., 2002; Gao et al., 2006; Pedersen et al., 2011; Zhang N. et al., 2011; Gao et al., 2012; Fan et al., 2016). A relative simple and rapid, *in vitro* refolding enzyme-linked immunosorbent assay (ELISA)-based method was able to discriminate between peptide-occupied and peptide-free SLA-I complexes based on monoclonal antibody PT85A binding (Oleksiewicz et al., 2002), because the PT85A monoclonal antibody can recognize all SLA class I molecules, from outbred as well as inbred pigs, and the conformational epitope was recognized by PT85A, required the presence of the ‘correct’ peptide as well as the ‘correct’ SLA class I molecule sequence (Lunney, 1994; Davis et al., 2000; Mosaad et al., 2006). We therefore selected seven SLA-1 molecules identified in Chinese Bama miniature pigs, and one CTL epitope peptide of classical swine fever virus (CSFV) and four epitope peptides of porcine reproductive and respiratory syndrome virus (PRRSV), previously identified by bioinformatics and immunological tests, for the current study. We aimed to construct SLA class I complexes consisting of viral epitope peptides, the extracellular region of the SLA-1 molecules, and β2m using splicing overlap extension polymerase chain reaction (SOE-PCR). The constructed protein complexes were then induced and expressed using an *Escherichia coli* prokaryotic expression system *in vitro*, and the obtained proteins were purified and refolded. Specific binding of SLA-1 proteins to the viral CTL epitope peptides was detected using an ELISA-based method and the MHC restrictions of the five epitope peptides were identified. The SLA-1 molecules selectively binding to an appropriate epitope peptide during antigen presentation were thus characterized *in vitro*. The results of this study will lay the foundations for further studies of cellular immune mechanisms and for the development of effective polypeptide vaccines.

## Materials and Methods

### Cloning of *SLA-1* Gene

Seven *SLA-1* alleles, including *SLA-1*^∗^0401, 1201, 1301, 11bm01, bm02, 08bm03, and 1101bm05, were obtained from Chinese Bama miniature pigs and submitted to the IPD-MHC database (Gao et al., 2014). The SLA Nomenclature Committee currently adopts the naming protocol for human leukocyte antigen alleles, and these alleles have therefore been renumbered as *SLA-1*^∗^04:01:01, 12:01, 13:01, 11:10, 20:01, 08:09, and 11:01:02, respectively, in the IPD-MHC database [Release 2.1.0.3 (2017-01-25)]. The seven alleles were cloned into the pMD 18-T vector (TaKaRa, Dalian, China) and the recombinant plasmids pMD18-T-SLA-1^∗^X (where X indicates 04:01:01, 12:01, 13:01, 11:10, 20:01, 08:09, or 11:01:02 allele) were collected for further study.

### Construction of Recombinant pET-SLA-1^∗^X-β2m and pET-Epitope-SLA-1^∗^X-β2m Expression System

**FIGURE 1:**
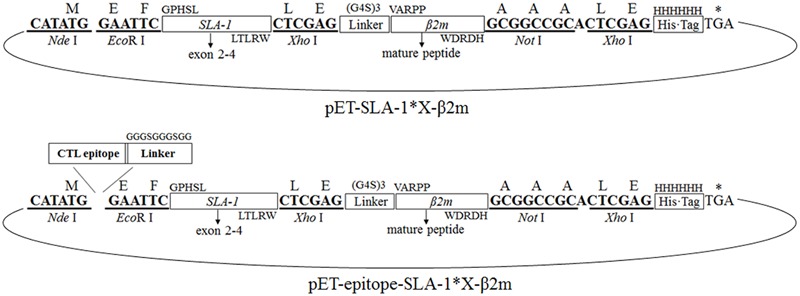
Construction of the pET-SLA-1^∗^X-β2m and pET-epitope-SLA-1^∗^X-β2m expression system. Horizontal boxes represent the amplified *SLA-1*^∗^X gene and *β2m* gene sequence, the glycine-rich linker gene sequence, the C-terminal His-tag sequence derived from the pET-30a(+) prokaryotic expression vector, and viral CTL epitope peptide sequence. Upper case letters outside boxes indicate amino acids. Bold letters indicate restriction enzymes sites. Arc represents the pET-30a(+) vector.

Seven *SLA-1* gene fragments encoding the extracellular domains (α1, α2, and α3 domains) were amplified from the plasmids pMD18-T-SLA-1^∗^X using primers P1aF/P1bF and P2R, respectively (**Table 1**). The swine *β2m* gene (complete mature β2m, omitting leader peptide) was amplified from cDNA from peripheral blood mononuclear cells of Chinese Bama miniature pig using primers P3F and P4R (**Table 1**). PCR amplifications were performed using KOD-Plus-Neo DNA polymerase system (Toyobo, Japan) according to the manufacturer’s instructions. The amplified *SLA-1*^∗^04:01:01 allele fragment and *β2m* gene were linked with a glycine-rich linker gene consisting of 15 amino acids (G4S)3 using the SOE-PCR method (Gao et al., 2006) (**Figure 1**). The SOE-PCR product was purified, ligated to the pMD18-T vector (TaKaRa), and sequenced. The verified fusion gene, named pMD18-T-SLA-1^∗^040101-β2m, was used as template to construct the remaining six recombinant genes, pMD18-T-SLA-1^∗^X-β2m, through restriction digestion with *Eco*R I and *Xho* I, ligation, and sequencing. The seven pMD18-T-SLA-1^∗^X-β2m fusion genes were then digested with *Nde* I and *Not* I and cloned into the pET-30a(+) prokaryotic expression vector at the *Nde* I and *Not* I restriction sites to create pET-SLA-1^∗^X-β2m.

**Table 1:** Splicing overlap extension PCR primers.

Name	Primer sequence (5′-3′)^a^	Amplified gene	Comments
P1aF	CGC **CATATG GAATTC** GGTCCCCACTCCCTGAGCTATTTC	04:01:01, 12:01, 13:01, 20:01, 11:01:02	Extracellular part of SLA-1, amino acids 22–295 (exons 2, 3, and 4).
P1bF	TCA **CATATG GAATTC** GGTCCCCACTCCCTGAGGTATTTC	11:10, 08:09	
P2R	ACCGCCAGAGCCACCTCCGCCTGAACCGCCTCCACC **CTCGAG** CCATCTCAGGGTGAGGGGCTCC	All *SLA-1* alleles	
P3F	GGTTCAGGCGGAGGTGGCTCTGGCGGTGGCGGATCG GTCGCGCGTCCCCCGAAGGTTC	*β*2m gene	Swine *β2m* gene (GenBank accession number NM213978), amino acids 21–118.
P4R	TT **GCGGCCGC** GTGGTCTCGATCCCACTTAACTATC		

*^a^Underlined part denotes a glycine-rich linker gene (G4S)3. Bold letters indicate restriction enzymes sites.*

One CTL epitope peptide of the CSFV non-structural protein 4A (NS4A) and four CTL epitope peptides of PRRSV glycoproteins 4 (GP4) and 5 (GP5), and the nucleocapsid protein (N), previously identified by bioinformatics and immunological tests (Pauly et al., 1995; Vashisht et al., 2008; Díaz et al., 2009), were selected to identify their MHC restriction. Double-stranded protein constructs consisting of selected viral CTL epitope peptides linked to the SLA-1 and β2m were constructed from single-stranded nucleotide sequences encoding the epitope peptides and the glycine-rich linker with *Nde* I and *Eco*R I compatible overhang (**Table 2**). The double-stranded nucleotides were generated by mixing equal amounts of each plus-sense and minus-sense nucleotide, heating to 95°C for 5 min, annealing at 37°C for 1 h, and cooling slowly to room temperature. The double-stranded constructs were digested with *Nde* I and *Eco*R I and ligated to pET-SLA-1^∗^X-β2m at the *Nde* I and *Eco*R I restriction sites, named pET-epitope-SLA-1^∗^X-β2m (‘epitope’ indicates the five selected viral CTL epitope peptides, respectively), as shown in **Figure 1**. All recombinant plasmids were verified by sequencing and restriction digestion and transfected into *E. coli* Rosetta (DE3).

**Table 2:** Nucleotides encoding viral CTL epitope peptides and the glycine-rich linker gene.

Virus	Protein	Sequence (5′-3′)^a^	Reference
		E N A L L V A L F	
CSFV	NS4A	NS4A-P: **TATG** GAA AAC GCT CTG CTG GTT GCT CTG TTC GGTGGCGGTTCCGGCGGTGGCTCCGGCGGT **G**	Pauly et al., 1995
		NS4A-M: **AATTC** ACCGCCGGAGCCACCGCCGGAACCGCCACC GAACAGAGCAACCAGCAGAGCGTTTTC **CA**	
		C L F A I L L A I	
PRRSV	GP4	GP4-5P: **TATG** TGT CTT TTT GCC ATC CTA CTG GCA ATT GGTGGCGGTTCCGGCGGTGGCTCCGGCGGT **G**	Díaz et al., 2009
		GP4-5M: **AATTC** ACCGCCGGAGCCACCGCCGGAACCGCCACC AATTGCCAGTAGGATGGCAAAAAGACA **CA**	
		N P E K P H F P L	
	N	N-15P: **TATG** AAC CCG GAG AAG CCC CAT TTC CCT CTA GGTGGCGGTTCCGGCGGTGGCTCCGGCGGT **G**	
		N-15M: **AATTC** ACCGCCGGAGCCACCGCCGGAACCGCCACC TAGAGGGAAATGGGGCTTCTCCGGGTT **CA**	
		F S L P T Q H T V	
		N-6P: **TATG** TTT AGT TTG CCG ACG CAA CAT ACT GTG GGTGGCGGTTCCGGCGGTGGCTCCGGCGGT **G**	
		N-6M: **AATTC** ACCGCCGGAGCCACCGCCGGAACCGCCACC CACAGTATGTTGCGTCGGCAAACTAAA **CA**	
		L A A L I C F V I R L A K N C	
	GP5	G9-P: **TATG** CTG GCT GCG CTG ATT TGC TTT GTC ATT AGG CTT GCG AAG AAC TGC	Vashisht et al., 2008
		GGTGGCGGTTCCGGCGGTGGCTCCGGCGGT **G**	
		G9-M: **AATTC** ACCGCCGGAGCCACCGCCGGAACCGCCACC GCAGTTCTTCGCAAGCCTAATGACAAAGC	
		AAATCAGCGCAGCCAG **CA**	

*^a^P and M indicate plus-sense and minus-sense sequences, respectively. Bold letters represent the sequences sticky to the ends of *Nde* I and *Eco*R I, respectively. Underlined part denotes the glycine-rich linker gene (**Figure 1**). The amino acid sequences of viral CTL epitope peptides are given above the plus-sense sequences.*

### Protein Expression, Sodium Dodecyl Sulfate–Polyacrylamide Gel Electrophoresis (SDS–PAGE), Refolding, and Western Blotting

Protein expression was performed as described previously (Liu et al., 2008). The harvested bacterial pellets were saved at -80°C for at most one day. Samples were analyzed by SDS-PAGE before and after induction with isopropyl β-D-1-thiogalactopyranoside (IPTG). All the recombinant proteins were present as insoluble inclusion bodies. Refolding of the recombinant protein in inclusion body form was carried out using a protein refolding kit (70123-3, Merck-Millipore, Germany) according to the manufacturer’s protocol. The refolded protein bands were electrotransferred onto polyvinylidene difluoride membranes and subjected to western blot analysis. The membrane was blocked overnight at 4°C with phosphate-buffered saline (PBS) containing 5% skim milk, incubated for 1 h at 37°C with 1:3000 diluted monoclonal anti-polyHistidine antibody (Sigma–Aldrich, United States), and washed three times for 5 min each with PBS containing 0.05% Tween 20 (PBST). The membrane was then incubated for 1 h at 37°C with horseradish peroxidase-conjugated goat anti-mouse IgG (H+L) antibody (Sigma–Aldrich) diluted at 1:10,000, and washed again three times. Proteins were visualized using 3,3′-diaminobenzidine/H_2_O_2_.

### Specific Binding of Viral CTL Epitope Peptides to SLA-1 Proteins

The specific binding of SLA-1 to viral CTL epitope peptides was analyzed using an ELISA-based method, as described previously (Oleksiewicz et al., 2002). Briefly, 100 μl of PBS containing 7.2 μg refolded protein was added to a single well of nickel-coated plates (Pierce, Thermo Fisher, United States) and used to capture the specific recombinant protein. The plates were shaken overnight at room temperature and washed three times with PBST for 5 min each. Monoclonal antibody PT85A (100 μl) (Monoclonal Antibody Center, Washington State University, United States) diluted 1:200 in 1% bovine serum albumin was then added to each well and the plates were incubated for 1 h at 37°C. Following washing, 100 μl/well of horseradish peroxidase-conjugated goat anti-mouse IgG (H+L) antibody (Sigma–Aldrich) diluted 1:10,000 in 1% bovine serum albumin was added and the plates were incubated for 1 h at 37°C. Finally, the plates were washed and developed for 20 min at room temperature with TMB (Sigma–Aldrich). The optical density (OD) was read at 450 nm using a standard microplate reader. Recombinant pET-SLA-1^∗^X-β2m protein was used as a negative control. The specific binding of SLA-1 proteins to viral CTL epitope peptides was determined by a relative OD values ≥ 2, calculated as OD (pET-epitope-SLA-1^∗^X-β2m)/OD (pET-SLA-1^∗^X-β2m). Each sample was assayed in triplicate to determine the mean value.

### Amino Acid Alignment of α1 and α2 Domains and PBG Comparison of SLA-1^∗^04:01:01 and SLA-1^∗^13:01 Molecules

The amino acid sequences of the α1 and α2 domains of the SLA-1 were compared using the search similarity and multiple alignment programs of the Lasergene package (DNASTAR, Madison, WI, United States). The crystal structure of the SLA-1^∗^04:01:01 molecule was obtained from the Protein Data Bank, with accession number 3QQ4 (Zhang N. et al., 2011). The 3D structure of the SLA-1^∗^13:01 molecule was modeled using SWISS-MODEL program^2^. All 3D structures were drawn using Chimera software^3^.

### Specific Binding of CSFV NS4A Epitope Peptide to Mutant SLA-1^∗^04:01:01 and SLA-1^∗^13:01 Molecules

We investigated the functions of the amino acids located in the pockets of the PBG in the α1 and α2 domains of the SLA-1^∗^04:01:01 and SLA-1^∗^13:01 by mutating the amino acid residues of one molecule to the corresponding amino acid residues of the other molecule by site-directed mutagenesis, using overlap PCR. The primers used for mutation of the SLA-1^∗^04:01:01 and SLA-1^∗^13:01 are listed in **Table 3**. pET-NS4A-SLA-1^∗^040101-β2m and pET-NS4A-SLA-1^∗^1301-β2m plasmids were used as templates, respectively. The SOE-PCR primers were 5′-CGC CAT ATG GAA AAC GCT CTG CTG GTT GCT CTG-3′, and 5′-TTG CGG CCG CGT GGT CTC GAT CCC ACT TAA CTA TC-3′ (underlined letters denote *Nde* I and *Not* I restriction sites, respectively). The resulting products were purified and digested with *Nde* I and *Not* I and ligated to the pET-30a(+) vector at the *Nde* I and *Not* I restriction sites. All mutant plasmids were verified by sequencing and restriction digestion and expressed in *E. coli* Rosetta (DE3). Mutant proteins were expressed in inclusion bodies and further refolded, as described above. Specific binding of the CSFV NS4A epitope peptide to the mutant SLA-1^∗^04:01:01 and SLA-1^∗^13:01 proteins was analyzed using an ELISA-based method, as described previously (Oleksiewicz et al., 2002). In order to better analyze the strength of peptides binding to SLA-1^∗^04:01:01, SLA-1^∗^13:01 and their mutants, the relative levels of CSFV NS4A epitope peptide binding to SLA-1^∗^04:01:01, SLA-1^∗^13:01, and their mutant molecules were compared. Briefly, the relative level (strength) of SLA-1^∗^13:01 molecule binding to epitope peptide was designated as 100% and the strength of peptide binding to other SLA-1 molecules were showed by the ratio of their relative OD values compared with SLA-1^∗^13:01.

**Table 3:** Overlap PCR primers used for mutation of SLA-1^∗^04:01:01 and SLA-1^∗^13:01 molecules.

Molecule	Name	Primer sequence (5′-3′)^a^	Mutant amino acid	Mutant molecule name
SLA-1^∗^04:01:01	04:01:01/66F	ATCGGGAGACGCGG**AAA**GTCAAGGAAAC	66 (N→K)	SLA-1^∗^04:01:01/66
	04:01:01/66R	GTTTCCTTGAC**TTT**CCGCGTCTCCCGAT		
	04:01:01/70F	AATGTCAAGGAA**AAC**GCACAGACTTAC	70 (T→N)	SLA-1^∗^04:01:01/70
	04:01:01/70R	GTAAGTCTGTGC**GTT**TTCCTTGACATT		
	04:01:01/99F	TCCAGAGCATG**TTT**GGCTGCTACTTGGGA	99 (Y→F)	SLA-1^∗^04:01:01/99
	04:01:01/99R	TCCCAAGTAGCAGCC**AAA**CATGCTCTGGA		
	04:01:01/151F	TGGGAGGCGGCC**AAT**GAGGCGGAGCGTAGGA	151 (D→N)	SLA-1^∗^04:01:01/151
	04:01:01/151R	TCCTACGCTCCGCCTC**ATT**GGCCGCCTCCCA		
	04:01:01/152F	GGCCGAT**GTG**GCGGAGCGTAGGAGGAGCTA	152 (E→V)	SLA-1^∗^04:01:01/152
	04:01:01/152R	TAGCTCCTCCTACGCTCCGC**CAC**ATCGGCC		
	04:01:01/151/152F	TGGGAGGCGGCC**AATGTG**GCGGAGCGTAGGA	151-152 (DE→NV)	SLA-1^∗^04:01:01/151/152
	04:01:01/151/152R	TCCTACGCTCCGC**CACATT**GGCCGCCTCCCA		
SLA-1^∗^13:01	13:01/66F	ATGAGGAGACGCGG**AAT**GTCAAGGACAA	66 (K→N)	SLA-1^∗^13:01/66
	13:01/66R	TTGTCCTTGAC**ATT**CCGCGTCTCCTCAT		
	13:01/70F	AAAGTCAAGGAC**ACC**GCACAGACTTAC	70 (N→T)	SLA-1^∗^13:01/70
	13:01/70R	GTAAGTCTGTGC**GGT**GTCCTTGACTTT		
	13:01/99F	TCCAGAGCATG**TAC**GGCTGCTACTTGGGA	99 (F→Y)	SLA-1^∗^13:01/99
	13:01/99R	TCCCAAGTAGCAGCC**GTA**CATGCTCTGGA		
	13:01/151F	TGGGAGGCGGCC**GAT**GTGGCGGAGCGTAGGA	151 (N→D)	SLA-1^∗^13:01/151
	13:01/151R	TCCTACGCTCCGCCAC**ATC**GGCCGCCTCCCA		
	13:01/152F	GGCCAAT**GAG**GCGGAGCGTAGGAGGAGCTA	152 (V→E)	SLA-1^∗^13:01/152
	13:01/152R	TAGCTCCTCCTACGCTCCGC**CTC**ATTGGCC		
	13:01/151/152F	TGGGAGGCGGCC**GATGAG**GCGGAGCGTAGGA	151-152 (NV→DE)	SLA-1^∗^13:01/151/152
	13:01/151/152R	TCCTACGCTCCGC**CTCATC**GGCCGCCTCCCA		

*^a^Letters in boldface type represent mutated nucleotides, which changed the corresponding amino acids.*

### Specific Binding of PRRSV Epitope Peptides to SLA-1^∗^04:01:01/151/152 and SLA-1^∗^13:01/151/152 Mutant Proteins

Double-stranded nucleotides of the four PRRSV epitope peptides were made by mixing equal amounts of the plus-sense and minus-sense nucleotides (**Table 2**), heating to 95°C for 5 min, annealing at 37°C for 1 h, and cooling slowly to room temperature. The double-stranded constructs were digested with *Nde* I and *Eco*R I and ligated to pET-SLA-1^∗^040101/151/152-β2m and pET-SLA-1^∗^1301/151/152-β2m at the *Nde* I and *Eco*R I restriction sites, respectively. Similarly, the recombinant proteins were expressed in inclusion bodies and further refolded, as described above. The specific binding of the four PRRSV epitope peptides to SLA-1^∗^04:01:01/151/152 and SLA-1^∗^13:01/151/152 was analyzed using an ELISA-based method, as described previously (Oleksiewicz et al., 2002).

## Results

### Construction of pET-SLA-1^∗^X-β2m and pET-Epitope-SLA-1^∗^X-β2m Expression Systems

The extracellular part of seven SLA-1 molecules and the mature peptide part of the *β2m* gene were successfully amplified to single fragments of 879 and 340 bp, respectively. The sequences were identical to the previously published sequences (Nygard et al., 2007; Gao et al., 2014). The SLA-1^∗^X-β2m fusion genes were obtained using the P1a/P1b and P4R primers. Agarose gel electrophoresis revealed a specific band at about 1200 bp, in accordance with the expected size of 1192 bp. The pET-SLA-1^∗^X-β2m expression systems were constructed as illustrated in **Figure 1**. The structures of the plasmids were confirmed by digestion with *Nde* I and *Not* I, followed by sequencing. The sequencing results showed that the inserted genes were 1192 bp in length, and identical to the extracellular sequences of SLA-1 and the mature peptide sequence of the *β2m* gene described above, with an insertion encoding a 15-amino acid glycine-rich linker. Similar results were found for the pET-epitope-SLA-1^∗^X-β2m expression systems (**Figure 1**). The inserted genes were 1249 bp (NS4A, GP4-5, N-15, and N-6 epitope peptides) and 1267 bp (G9 epitope peptide) in length, and identical to the epitope peptide sequences and SLA-1^∗^X-β2m fusion gene sequences, with an insertion encoding a 10 amino acid glycine-rich linker.

### Expression, SDS–PAGE, and Western Blotting

**FIGURE 2:**
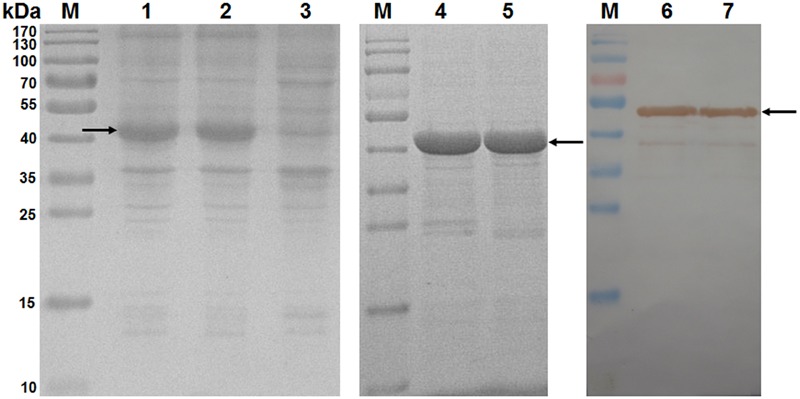
Analysis of inclusion body proteins by SDS-PAGE and western blotting. M: protein marker; lanes 1 and 2: pET-NS4A-SLA-1^∗^040101-β2m and pET-SLA-1^∗^040101-β2m proteins after induction with IPTG in *E. coli* Rosetta; lane 3: pET-NS4A-SLA-1^∗^040101-β2m protein before induction with IPTG; lanes 4 and 5: purified and refolded pET-NS4A-SLA-1^∗^040101-β2m and pET-SLA-1^∗^040101-β2m proteins; lanes 6 and 7: pET-NS4A-SLA-1^∗^040101-β2m and pET-SLA-1^∗^040101-β2m proteins visualized by western blotting. Arrows indicate expected position of recombinant proteins.

A total of 42 different recombinant proteins were expressed from the pET-30a(+) vector in *E. coli* Rosetta, including seven pET-SLA-1^∗^X-β2m proteins and 35 pET-epitope-SLA-1^∗^X-β2m proteins (‘epitope’ indicates NS4A, GP4-5, N-15, N-6, or G9 epitope peptide, X indicates 04:01:01, 12:01, 13:01, 11:10, 20:01, 08:09, or 11:01:02 molecule). All recombinant protein products were inducible with 1 mmol/L IPTG, and were not produced in non-induced cultures (**Figure 2**, lane 3). For example, SDS-PAGE showed that the transformed cells with pET-SLA-1^∗^040101-β2m produced a large amount of a protein with a mass of about 45 kDa (**Figure 2**, lane 2), and analysis of pET-NS4A-SLA-1^∗^040101-β2m showed protein expression at a position equivalent to a mass of about 47 kDa (**Figure 2**, lane 1). Similar results were found for the other recombinant proteins. Solubility analysis showed that all the recombinant proteins were present as insoluble inclusion bodies. Effective inclusion body purification and protein refolding produced activated protein preparations of adequate purity (**Figure 2**, lane 4 and 5). Western blotting with an anti-His tag monoclonal antibody showed that the vector-encoded C-terminal His-tag was present in all the recombinant proteins (**Figure 2**, lane 6 and 7).

### Detection of Specific Binding of Viral CTL Epitope Peptides to SLA-1 Proteins

**FIGURE 3:**
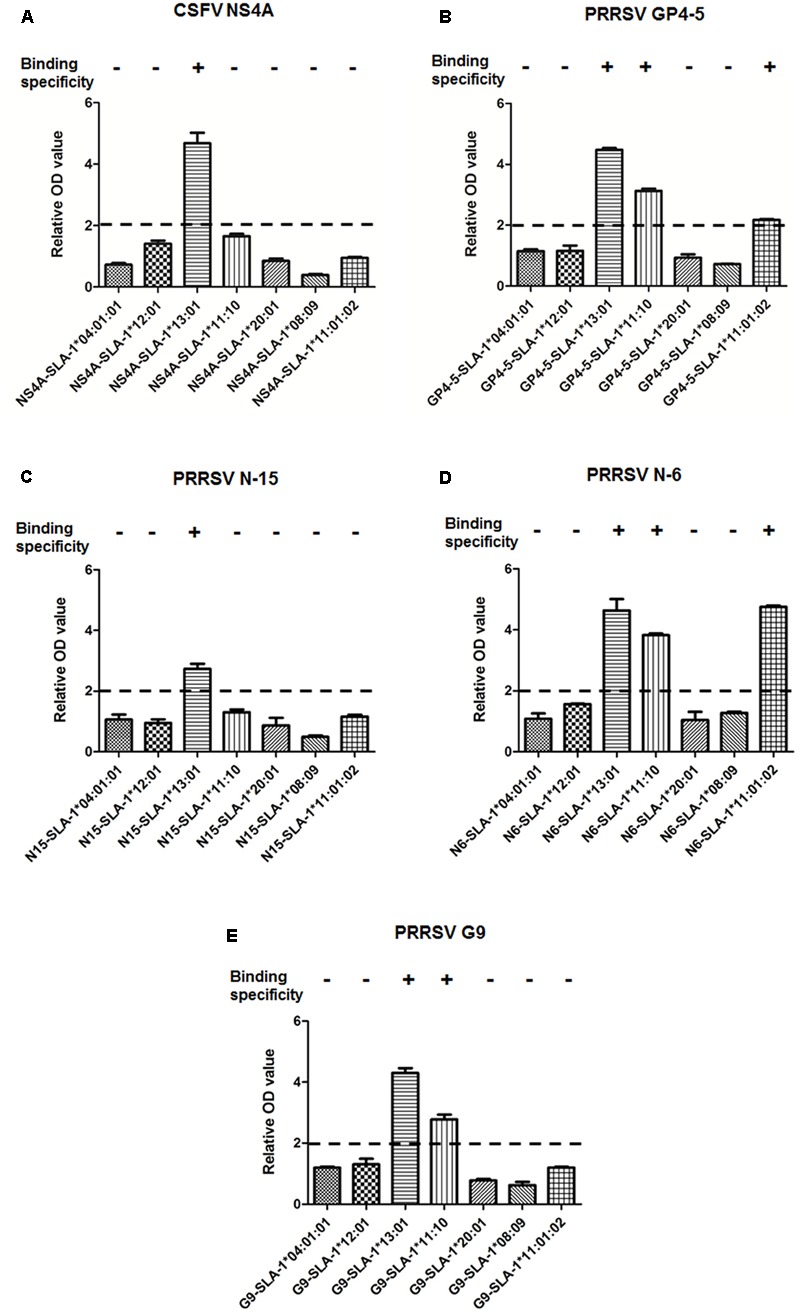
Specific binding of viral CTL epitope peptides to SLA-1 proteins. Specific binding of SLA-1 to viral CTL epitope peptides was determined by a relative OD value ≥ 2. Each sample was assayed in triplicate to determine the mean value. **(A)** Specific binding of seven SLA-1 proteins to CSFV NS4A epitope peptide. **(B)** Specific binding of seven SLA-1 proteins to PRRSV GP4-5 epitope peptide. **(C)** Specific binding of seven SLA-1 proteins to PRRSV N-15 epitope peptide. **(D)** Specific binding of seven SLA-1 proteins to PRRSV N-6 epitope peptide. **(E)** Specific binding of seven SLA-1 proteins to PRRSV G9 epitope peptide.

The results of detection using the ELISA-based method are illustrated in **Figure 3** (**Supplementary Table S1**). SLA-1^∗^13:01, SLA-1^∗^11:10, and SLA-1^∗^11:01:02 bound specifically to different CTL epitopes of CSFV and PRRSV, and the MHC restrictions of the five epitopes were identified. For example, the pET-NS4A-SLA-1^∗^13:01-β2m protein exhibited 4.7-fold higher reactivity than pET-SLA-1^∗^13:01-β2m, and the relative OD values for the other SLA-1 were < 2.0 (**Figure 3A**). This demonstrated that there was specific binding of the CSFV NS4A epitope peptide to the SLA-1^∗^13:01; i.e., the CSFV NS4A (ENALLVALF) peptide was a SLA-1^∗^13:01-restricted CTL epitope. Similarly, the PRRSV N-15 (NPEKPHFPL) peptide was a SLA-1^∗^13:01-restricted CTL epitope (**Figure 3C**). The MHC restrictions of PRRSV GP4-5 (CLFAILLAI) and N-6 (FSLPTQHTV) epitopes were identical, and they could bind specifically to SLA-1^∗^13:01, SLA-1^∗^11:10, and SLA-1^∗^11:01:02 (**Figures 3B,D**). The PRRSV G9 (LAALICFVIRLAKNC) peptide bound specifically to SLA-1^∗^13:01 and SLA-1^∗^11:10 and was thus a SLA-1^∗^13:01- and SLA-1^∗^11:10-restricted CTL epitope (**Figure 3E**).

### Amino Acid Sequence Analysis and PBG Comparison

**FIGURE 4:**
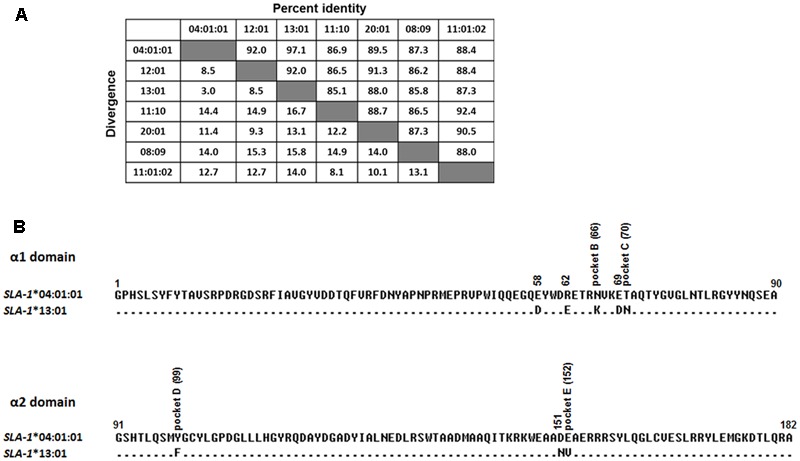
Amino acid sequence homology of α1 and α2 domains among seven SLA-1 molecules **(A)** and amino acid alignment of α1 and α2 domains between SLA-1^∗^04:01:01 and SLA-1^∗^13:01 molecules **(B)**. Numbers above the line indicate the amino acid sequence numbers of each sequence counted from the α1 domain of the SLA-1 molecule. Dots indicate identical amino acids compared with the SLA-1 molecules common consensus sequence.

**FIGURE 5:**
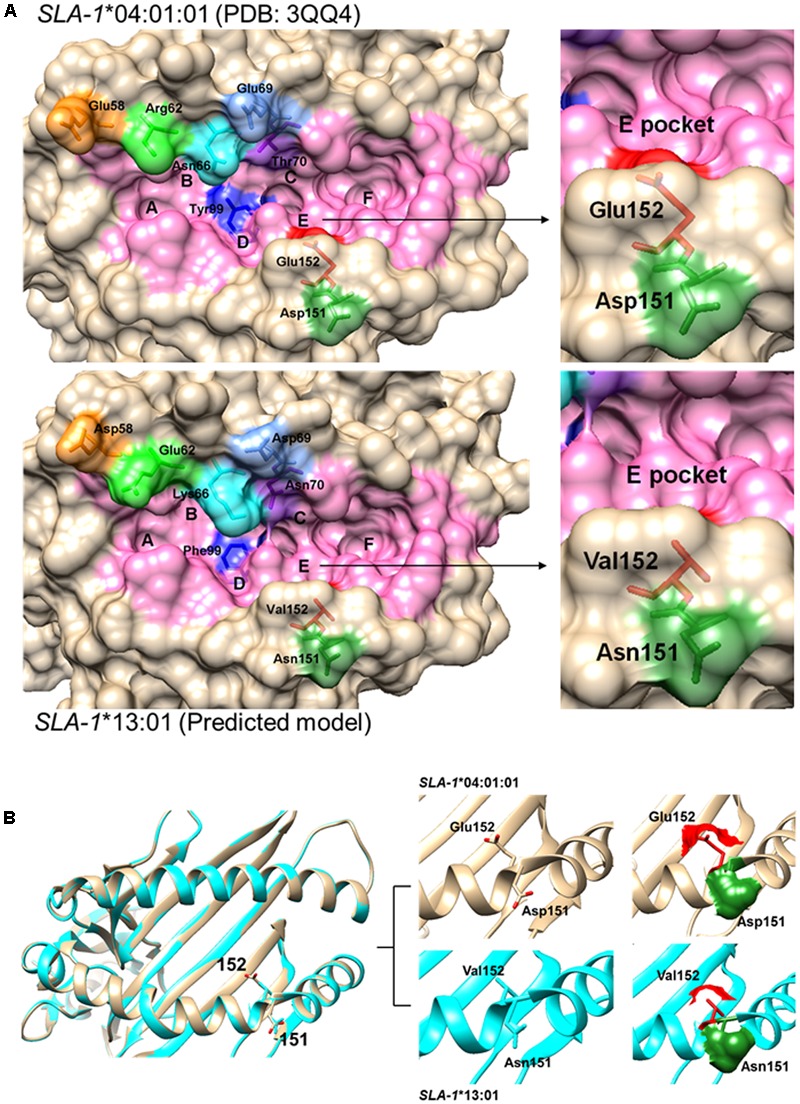
Structural alignments of the PBG of SLA-1^∗^04:01:01 and SLA-1^∗^13:01 molecules. **(A)** 3D models of SLA-1^∗^04:01:01 (PDB code: 3QQ4) and SLA-1^∗^13:01 built by the SWISS-MODEL program. The PBG is shown in pink. Eight discrepant amino acid residues are indicated by different colors in the stick model. **(B)** Superimposition of SLA-1^∗^04:01:01 and SLA-1^∗^13:01 PBG structures showing conformational variation in Asp^151^Glu^152^ and Asn^151^Val^152^. SLA-1^∗^04:01:01 is shown in light brown and SLA-1^∗^13:01 in cyan. The stick models and surfaces of residues at positions 151 and 152 are shown in green and red, respectively.

Analysis of the amino acid sequence homologies of the α1 and α2 domains among the seven SLA-1 molecules showed the highest homology between SLA-1^∗^04:01:01 and SLA-1^∗^13:01, of up to 97.1% (**Figure 4A**). Alignment of the amino acid sequences of the α1 and α2 domains showed that only eight amino acid residues, 58(E/D), 62(R/E), 66(N/K), 69(E/D), 70(T/N), 99(Y/F), 151(D/N), and 152(E/V), were discrepant between the SLA-1^∗^04:01:01 and SLA-1^∗^13:01 molecules (**Figure 4B**). The 3D structure of the SLA-1^∗^13:01 molecule was modeled using the SWISS-MODEL program based on the crystal structure of the SLA-1^∗^04:01:01 molecule (Zhang N. et al., 2011), and the PBGs of SLA-1^∗^04:01:01 and SLA-1^∗^13:01 were compared. Four discrepant residues were involved in constituting six pockets of the PBG: pocket B [66(N/K)], pocket C [70(T/N)], pocket D [99(Y/F)], and pocket E [152(E/V)] (**Figure 5**). The other four discrepant residues might play roles in the support, stability, and connection of the six pockets.

### Detection of Specific Binding of CSFV NS4A Epitope Peptide to Mutant SLA-1^∗^04:01:01 and SLA-1^∗^13:01 Proteins

**FIGURE 6:**
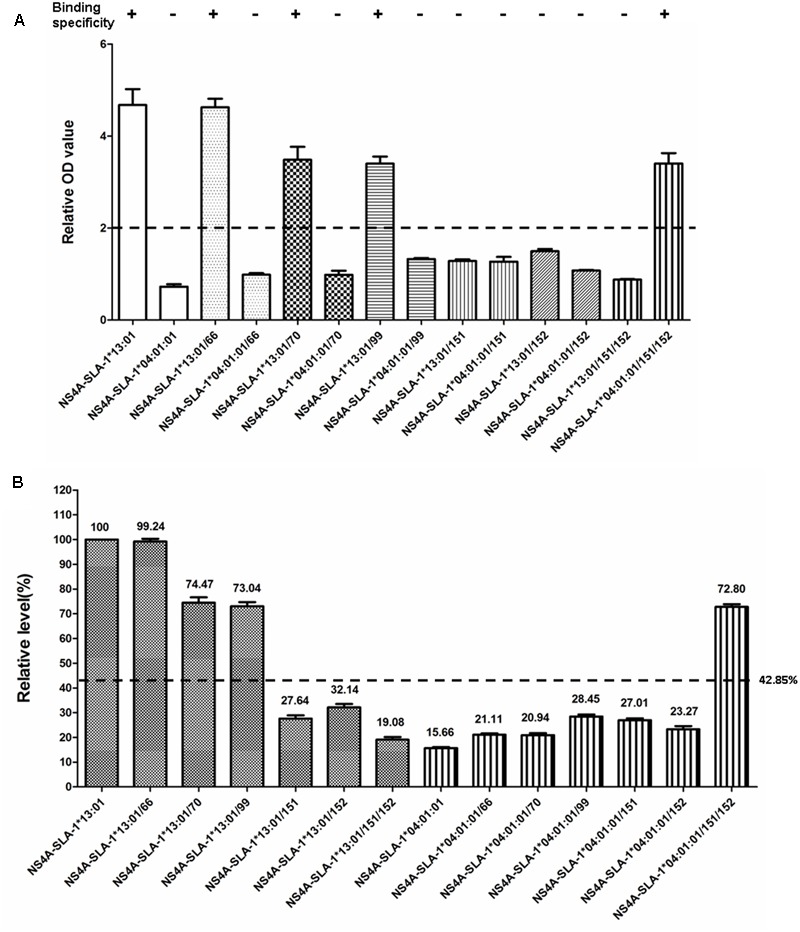
Specific binding of CSFV NS4A epitope peptide to mutant SLA-1^∗^04:01:01 and SLA-1^∗^13:01 proteins. **(A)** Detection of specific binding of NS4A peptide to mutant SLA-1^∗^04:01:01 and SLA-1^∗^13:01 proteins. Specific binding of the mutant SLA-1 proteins to the epitope peptide were determined by a relative OD value ≥ 2. Columns of the same pattern indicate the results of NS4A epitope peptide-specific binding to SLA-1^∗^04:01:01 and SLA-1^∗^13:01 proteins with mutated residues at the same position, respectively. **(B)** The binding strength of NS4A peptide to mutant SLA-1^∗^04:01:01 and SLA-1^∗^13:01 proteins. The relative level (strength) of SLA-1^∗^13:01 molecule binding to epitope peptide was designated as 100% and the strength of peptide binding to other SLA-1 molecules were showed by the ratio of their relative OD values compared with SLA-1^∗^13:01.

SLA-1^∗^04:01:01 and SLA-1^∗^13:01 molecules showed high amino acid sequence homology (97.1%) in the α1 and α2 domains, but exhibited different binding specificities to viral epitopes (**Figure 3**). We therefore mutated four discrepant residues in one molecule, 66(N/K), 70(T/N), 99(Y/F), and 152(E/V), respectively, to the corresponding residues of the other molecule and analyzed the effects on their binding specificity to the CSFV NS4A epitope peptide (**Supplementary Table S2**). The relative OD values were > 2.0 when the Lys^66^, Asn^70^, and Phe^99^ residues of the SLA-1^∗^13:01 were mutated, respectively (**Figure 6A**). SLA-1^∗^13:01/66, SLA-1^∗^13:01/70, and SLA-1^∗^13:01/99 mutant proteins retained specific binding to the CSFV NS4A epitope peptide, but specific binding was lost in the SLA-1^∗^13:01/152 mutant protein with a mutated Val^152^ residue. In the case of the SLA-1^∗^04:01:01, the NS4A epitope peptide could not bind to any of the four mutant proteins in which the Asn^66^, Thr^70^, Tyr^99^, or Glu^152^ residue was mutated to the corresponding amino acid residue of the SLA-1^∗^13:01 (**Figure 6A**). Similar situations were found when the 151(D/N) residue of one molecule was mutated to the corresponding amino acid residue of the other molecule. However, when the 151(D/N) and 152(E/V) residues of SLA-1^∗^13:01 and SLA-1^∗^04:01:01 were mutated simultaneously, the NS4A epitope peptide could still bind to the SLA-1^∗^04:01:01/151/152 mutant protein, but not to the SLA-1^∗^13:01/151/152 mutant protein (**Figure 6A**). In addition, the binding strength of NS4A peptide to SLA-1^∗^13:01 molecule were decreased at different degrees when mutating these amino acid residues to the corresponding residues of SLA-1^∗^04:01. However, the binding strength of NS4A peptide to SLA-1^∗^04:01:01/151/152 mutant was obviously increased compared with SLA-1^∗^04:01:01 and was slightly lower than SLA-1^∗^13:01 (**Figure 6B**).

### Detection of Specific Binding of PRRSV Epitope Peptides to SLA-1^∗^04:01:01/151/152 and SLA-1^∗^13:01/151/152 Mutant Proteins

**FIGURE 7:**
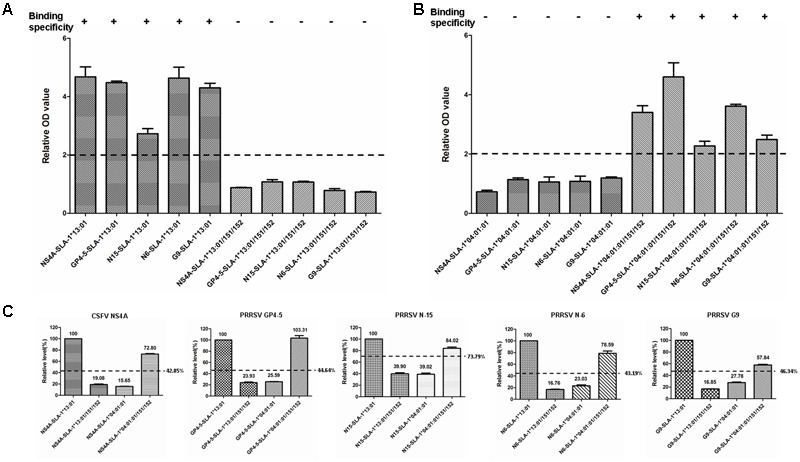
Specific binding of five viral epitope peptides to SLA-1^∗^04:01:01/151/152 and SLA-1^∗^13:01/151/152 mutant proteins. **(A)** Detection of specific binding of five viral epitope peptides to SLA-1^∗^13:01 when the residues at positions 151 and 152 were simultaneously mutated to the corresponding amino acid residues of the SLA-1^∗^04:01:01. **(B)** Detection of specific binding of five viral epitope peptides to SLA-1^∗^04:01:01 when residues at positions 151 and 152 were simultaneously mutated to the corresponding amino acid residues of the SLA-1^∗^13:01. **(C)** The binding strength of five viral epitope peptides to SLA-1^∗^04:01:01/151/152 and SLA-1^∗^13:01/151/152 mutant proteins. The relative levels (strength) of SLA-1^∗^13:01 molecule binding to epitope peptide were designated as 100% and the strength of peptide binding to other SLA-1 molecules were showed by the ratio of their relative OD values compared with SLA-1^∗^13:01.

The specific binding of the four PRRSV epitope peptides (GP4-5, N-15, N-6, and G9) to the SLA-1^∗^04:01:01/151/152 and SLA-1^∗^13:01/151/152 mutant proteins are shown in **Figure 7** (**Supplementary Table S3**). Similar to the results for the CSFV NS4A epitope peptide, the PRRSV GP4-5, N-15, N-6, and G9 epitope peptides could bind specifically to the SLA-1^∗^04:01:01/151/152 mutant protein, but not to the SLA-1^∗^13:01/151/152 mutant protein. Similarly, the binding strength of SLA-1^∗^04:01:01/151/152 mutant to the four epitope peptides had obvious increasing compared with SLA-1^∗^04:01:01 and was slightly lower than SLA-1^∗^13:01 except GP4-5 peptide. In turn, the binding strength of SLA-1^∗^13:01/151/152 mutant to four epitope peptides were nearly identical to SLA-1^∗^04:01:01. We therefore speculated that the fixed combination of amino acid residues at positions 151 and 152 (Asn^151^Val^152^) might be the key residues affecting the binding of viral CTL epitope peptides to SLA-1^∗^13:01 and SLA-1^∗^04:01:01. Structural alignment of the PBGs of SLA-1^∗^13:01 and SLA-1^∗^04:01:01 revealed that pocket E was larger in SLA-1^∗^13:01 than in SLA-1^∗^04:01:01 as a result of different surfaces of the concave Val^152^ and salient Glu^152^ residues (**Figure 5A**). Simultaneously, the Asn^151^ residue of SLA-1^∗^13:01 occurred in turn and random coil content and presented more flexibility than Asp^151^ of SLA-1^∗^04:01:01 to support pocket E (**Figure 5B**). Pocket E in the SLA-1^∗^13:01 protein might thus have fewer steric limitations and be able to accommodate more residues of viral CTL epitope peptides.

## Discussion

Three constitutively expressed and highly polymorphic classical SLA class I genes map to the SLA complex, *SLA-1, SLA-2*, and *SLA-3* (Renard et al., 2006). SLA class I molecules play a crucial role in cellular immune antigen presentation in pigs and in xenotransplantation of pig organs into humans. The *SLA-1* gene has the highest expression level (Tennant et al., 2007), implying that SLA-1 molecules might play a dominant role in the immune process, including presentation of CTL epitopes. Sixty-nine *SLA-1* alleles, identified in various pig breeds and cell lines (Smith et al., 2005; Lee et al., 2005, 2008; Ho et al., 2006, 2009, 2010; Gao et al., 2014, 2017), have been deposited in the IPD-MHC SLA sequence database to date, and this number is increasing as more efficient SLA-typing techniques are employed and more experimental pig breeds are developed worldwide. Polymorphism of SLA class I molecules is concentrated in the region of the PBG, and determines the distinct structure of the PBG. The structural basis of swine CTL epitope presentation has been illustrated by determination of the crystal structure of SLA-1^∗^04:01:01 with a peptide from swine influenza A virus and Ebola virus (Zhang N. et al., 2011). The PBG was shown to be classified into six pockets, A–F, which determined the peptide-binding specificity. The binding of different SLA molecules to different classes of peptides was determined by the fit between these pockets and the anchor residues in the peptides (Zhang N. et al., 2011; Fan et al., 2016). Viral peptides interact with the pockets to form a heterotrimeric complex, including SLA class I heavy chain, the epitope peptide, and β2m. In the initial stage of virus infection, antigenic proteins are processed in a proteasome-dependent or -independent manner, and the resultant short peptides are then transported to the endoplasmic reticulum and loaded onto the PBG of SLA class I molecules. The peptide-loaded SLA class I complexes are then translocated to the cell surface and recognized by CTLs with specific T-cell receptors (Shastri et al., 2002; Yewdell and Haeryfar, 2005). This immune recognition induces an MHC-restricted CD8+ T-cell response characterized by the proliferation of CTLs, prevention of pathogen release, lysis of the virally infected cells, and killing and elimination of the infected cells by host effector T cells (Neefjes et al., 2011). Identification of epitope peptide binding to SLA class I molecules represents a critical step in this process. Moreover, viral epitopes bind to the PBG with different specificities, in an MHC-restricted manner. It is therefore essential to characterize the binding of SLA class I molecules to viral CTL epitope peptides because of the significance of this process for monitoring CD8+ T cell immune responses and understanding the mechanisms of cellular immunity.

Historically, the peptide-binding specificity of SLA class I molecules has been characterized by various methods, each with particular advantages and drawbacks (Oleksiewicz et al., 2002; Shastri et al., 2002; Sylvester-Hvid et al., 2002; Gao et al., 2006; Pedersen et al., 2011; Zhang N. et al., 2011; Pedersen et al., 2014, 2016). The refolding of the MHC class I complex is known to be influenced by the presence or absence of a peptide (Garboczi et al., 1992). The refolding and conformation of the SLA class I complex depends on whether the epitope peptide can bind to the SLA class I molecules. Epitope has generally been reported to be required for *in vitro* refolding of SLA class I molecules (Garboczi et al., 1992). The refolding and conformational differences can be assessed using the monoclonal antibody PT85A, and differences in binding reactivities between epitope peptides and SLA-1 molecules can then be reflected by the relative OD values following ELISA. In this study, we used a rapid and simple *in vitro* refolding assay (Oleksiewicz et al., 2002) to examine the specific binding of seven SLA-1 proteins to five viral CTL epitope peptides, previously identified using bioinformatics and immunological tests (Pauly et al., 1995; Vashisht et al., 2008; Díaz et al., 2009), but with unknown MHC restriction. Although the CSFV NS4A epitope peptide (ENALLVALF) has been described as a SLA d/d haplotype (H4/H4 haplotype: *SLA-1*^∗^04:01:01-*SLA-2*^∗^04:01-*SLA-3*^∗^04:01)-restricted CD8+ T cell epitope (Pauly et al., 1995), it was unknown to which of these three class I genes it was restricted. The current results showed that SLA-1^∗^13:01, SLA-1^∗^11:10, and SLA-1^∗^11:01:02 proteins were able to bind specifically to different CTL epitopes of CSFV and PRRSV. This suggests that the compatibilities of the pockets in the PBG of SLA class I molecules are distinct, and that a broad range of protective CD8+ T cell responses could potentially be elicited, associated with the various peptide conformations. The distinct peptide conformations mainly result from the flexible side chains of the pockets, and do not involve the α-helixes and β-sheets that form the PBG. The fact that different SLA-1 can bind to the same CTL epitope indicates that the same peptide could be presented by different SLA class I molecules, with chemical specificity determined by different preferences for certain anchor residues. Many *SLA-1* suballeles could also show similar anchor residue preference due to the presence of identical key residues in pockets (Stewart-Jones et al., 2005). Many studies have also shown that water molecules, platform adjustment, or the presence of only one flexible pocket can contribute to the accommodation of different peptides (Smith et al., 1996; Stewart-Jones et al., 2005; Koch et al., 2007). Furthermore, although the conformations of one SLA-1 pockets are unique, similar pockets were could found in other SLA-1 molecule structures. These pockets could accommodate the same residues of one peptide in similar manners (Fan et al., 2016).

We identified the MHC restriction of five CTL epitopes. CD8+ T cell epitopes are known to be strictly restricted to SLA class I molecules, suggesting that identification of MHC-I-restricted CTL epitopes could aid the rational development and modification of peptide-based vaccines. Current vaccines are usually ineffective against newly emerged virus strains because of the rapid mutation of viral proteins through both antigenic drift and shift, for example influenza virus. New vaccines therefore need to be developed against their mutant virus, and new-vaccine strategies increasingly are directed at conserved viral CTL epitope-based vaccines. Few polypeptide vaccines have been utilized in swine to date because of the MHC restriction of peptides and the high polymorphism of SLA class I genes. In this study, we constructed SLA class I complexes consisting of viral epitope peptides, the extracellular region of SLA-1 molecules, and β2m, and expressed and refolded them *in vitro*, and used an ELISA-based method to determine the MHC restriction of five CTL epitopes. It is hoped to develop the more effective polypeptide vaccines for these CTL epitope peptides. Many T-cell epitopes derived from various swine-origin viruses have been identified using *in vivo* cytotoxicity assays, enzyme-linked immunospot assays, intracellular cytokine staining, flow cytometry, and so on (Pauly et al., 1995; Gerner et al., 2006; Vashisht et al., 2008; Díaz et al., 2009; Wang et al., 2011; Zhang W. et al., 2011; Chen et al., 2013), but most of these methods are relatively labor intensive and technically demanding, and importantly do not clarify the MHC restriction of the epitopes. Gao et al. reconstructed a SLA-2-(G4S)3-b2m protein complex *in vitro*, which could be used to identify nonameric viral peptides in swine, in conjunction with mass spectrometry (Gao et al., 2006). Similarly, the current study presented a method for not only generating recombinant SLA-1 molecules and mapping their specificities, but also for identifying nonameric MHC-I-restricted epitopes. This method could avoid the one-sidedness of predicting epitopes only depending on protein sequences and thus improve the accuracy of epitope identification.

In addition, we analyzed the amino acid sequence homologies of the α1 and α2 domains among seven SLA-1 molecules. The SLA-1^∗^04:01:01 and SLA-1^∗^13:01 had 97.1% homology, implying that they might bind the same classes of peptide. However, these proteins showed distinctly different epitope-binding specificities. We examined the basis for these different binding specificities by alignment of the amino acid residues of the PBG and observed eight discrepant amino acid residues, of which four [66(N/K), 70(T/N), 99(Y/F), 152(E/V)] were involved in the formation of pockets B, C, D, and E, respectively. We mutated each of the four different amino acid residues of one molecule to the corresponding residues of the other molecule and determined the effect of the mutations on the specific binding of the CSFV NS4A epitope peptide. The NS4A epitope peptide could not bind to the SLA-1^∗^13:01/152 mutant protein when Val^152^ alone was mutated, and could not bind to the SLA-1^∗^04:01:01/152 mutant protein. We obtained similar results for the Asn^151^ residue. However, when the 151(D/N) and 152(E/V) residues of SLA-1^∗^13:01 and SLA-1^∗^04:01:01 were mutated simultaneously to those of the corresponding molecule, the five epitope peptides could not bind to the SLA-1^∗^13:01/151/152 mutant protein, but could bind to the SLA-1^∗^04:01:01/151/152 mutant protein. Moreover, the relative level of epitope peptides binding to SLA-1^∗^04:01:01/151/152 mutant was obviously increased, indicating the binding strength of peptides to SLA-1^∗^04:01:01 was enhanced when simultaneously mutating 151 and 152 amino acid residues. In turn, the binding strength of SLA-1^∗^13:01/151/152 mutant to epitope peptides were nearly identical to SLA-1^∗^04:01:01, indicating the weak binding strength. This suggests that the fixed combination of Asn^151^Val^152^ residues might be the key residues influencing the binding of viral CTL epitope peptides to SLA-1^∗^13:01 and SLA-1^∗^04:01:01 proteins. The crystal structure of SLA-1^∗^04:01:01 revealed that Arg^156^ in pocket D had a ‘one-ballot veto’ function in peptide binding, due to its flexible side chain (Zhang N. et al., 2011). However, the different epitope peptide conformations are caused not only by the flexibility of the side chains of the residues in the PBG, but also by skewing of the α1 and α2 helixes forming the PBG (Fan et al., 2016). Thus although the Asn^151^ residue of SLA-1^∗^13:01 did not constitute part of the pockets of the PBG, it occurred in turn and random coil content and thus increased the flexibility support of pocket E, which may result in skewing of the α helixes forming the PBG. Furthermore, pocket E of SLA-1^∗^13:01 was larger than that of SLA-1^∗^04:01:01 as a result of the different surfaces of the concave Val^152^ and salient Glu^152^ residues, suggesting that pocket E in the SLA-1^∗^13:01 protein might have fewer steric limitations and be able to accommodate more viral CTL epitope peptides. The flexible pocket E of SLA-1^∗^04:01:01/151/152 might also adopt various conformations to accommodate the peptides through mutation of the Asp^151^ and Glu^152^ residues, leading to distinct peptide conformations, and might thus play critical biochemical roles in determining the peptide-binding motif of SLA-1^∗^13:01. A strong correlation exists between high-affinity SLA-I binding peptides and the stability of the complexes, with high-affinity SLA-I binding peptides generally forming more stable complexes (Pedersen et al., 2016). Changes in several amino acid residues of SLA class I molecules could thus influence the affinity and stability of complexes, thereby affecting the peptide-binding specificity of SLA class I molecules. Furthermore, the binding specificity also could be affected by changes in amino acid polarity. Interestingly, in contrast to a previous study (Oleksiewicz et al., 2002), we found that the CSFV NS4A epitope peptide could not bind to SLA-1^∗^04:01:01 but could bind to SLA-1^∗^13:01, with relative OD values of up to 4.7. However, the NS4A epitope peptide could bind to the SLA-1^∗^04:01:01/151/152 mutant protein when both the Asp^151^ and Glu^152^ residues were mutated simultaneously. This discrepancy may be because the *in vitro* refolding method for the protein complexes in the current study differed from that used in the previous study, and the binding specificity of the H4 w/o protein with synthetic (free) NS4A peptide was also not observed in the refold reactions (Oleksiewicz et al., 2002). Further work is therefore needed to determine the crystal structure of the NS4A peptide-SLA-1^∗^04:01:01 complex to thoroughly explore this phenomenon. Certainly, it is beneficial if the promiscuity of SLA-1^∗^13:01 and SLA-1^∗^04:01:01 were investigated in terms of the peptide binding affinity and the stability of the complexes. Therefore, our future study will focused on the peptide binding affinity and stability to SLA class I molecules and stability of the SLA complex by using various methods, for example, luminescent oxygen channeling assay and scintillation proximity assay-based peptide-SLA dissociation. Furthermore, we also will attempt to perform molecular dynamics simulations to investigate the binding difference of SLA class I molecular to epitope peptides by using various software, for example, Discovery Studio and CHARMM.

## Conclusion

We successfully constructed SLA class I complexes consisting of viral epitope peptides, the extracellular region of SLA-1 molecules, and β2m *in vitro*. We also detected the specific binding of seven SLA-1 proteins to five viral CTL epitope peptides through the expression and refolding of the protein complexes. The SLA-1^∗^13:01, SLA-1^∗^11:10, and SLA-1^∗^11:01:02 proteins were able to specifically bind different CTL epitopes of CSFV and PRRSV, and the MHC restrictions of five epitopes were identified. Moreover, the fixed combination of Asn^151^Val^152^ residues might represent the key residues influencing the binding of viral several CTL epitope peptides to SLA-1^∗^13:01 and SLA-1^∗^04:01:01. The increased flexibility of pocket E in the SLA-1^∗^13:01 protein might play a critical biochemical role in determining the peptide-binding motif of SLA-1^∗^13:01. Characterization of the peptide-specific binding properties of SLA class I molecules provides essential information for the future identification of novel epitopes, as well as for the overall validation and analysis of currently available or newly developed CTL-based vaccines in swine. This information may also lead to improved understanding of the structural basis of CTL-based immune responses.

## Author Contributions

CG, HC, and LQ designed the experiments, analyzed the data and wrote the paper. CG, XH, JQ, QJ, and HL performed the experiments.

## Conflict of Interest Statement

The authors declare that the research was conducted in the absence of any commercial or financial relationships that could be construed as a potential conflict of interest.

## Footnotes

1 http://www.ebi.ac.uk/ipd/mhc/group/SLA

2 http://swissmodel.expasy.org/

3 http://www.cgl.ucsf.edu/chimera/

## Supplementary Material

TABLE S1 The raw ELISA data of **Figure 3**.

TABLE S2 The raw ELISA data of **Figure 6**.

TABLE S3 The raw ELISA data of **Figure 7**.

## Abbreviations

3D: three-dimensional
CSFV: classical swine fever virus
CTL: cytotoxic T lymphocyte
ELISA: enzyme-linked immunosorbent assay
GP4: glycoprotein 4
GP5: glycoprotein 5
IPD: Immuno Polymorphism Database
IPTG: isopropyl β-D-1-thiogalactopyranoside
MHC: major histocompatibility complex
N: nucleocapsid
NK: natural killer
NS4A: non-structural protein 4A
OD: optical density.
PBG: peptide-binding groove
PBS: phosphate-buffered saline
PRRSV: porcine reproductive and respiratory syndrome virus
SDS-PAGE: sodium dodecyl sulfate-polyacrylamide gel electrophoresis
SLA: swine leukocyte antigen
SOE-PCR: splicing overlap extension polymerase chain reaction
β 2m: β 2-microglobulin
